# Reduced Brain Iron is Associated with Striatal Hyperdopaminergia in Schizophrenia and Unrelated to Neuromelanin and Myelin Imaging Measures: A Quantitative Susceptibility Mapping MRI and PET Study

**DOI:** 10.1176/appi.ajp.20240512

**Published:** 2025-09-01

**Authors:** Luke James Vano, Robert Ali McCutcheon, Jan Sedlacik, Stephen John Kaar, Grazia Rutigliano, Giovanna Nordio, Valeria Finelli, Leigh Townsend, Alaine Berry, Ben Statton, Amir Fazlollahi, Mattia Veronese, Seth Cabot Hopkins, Kenneth Stephen Koblan, Ian Paul Everall, Oliver David Howes

**Affiliations:** 1 Department of Psychosis Studies, Institute of Psychiatry, Psychology & Neuroscience, King’s College London, London, United Kingdom; 2 Psychiatric Imaging Group, MRC Laboratory of Medical Sciences, Hammersmith Hospital, London, United Kingdom; 3 Institute of Clinical Sciences, Faculty of Medicine, Imperial College London, London, United Kingdom; 4 South London and Maudsley NHS Foundation Trust, London, United Kingdom; 5 Department of Psychiatry, University of Oxford, Oxford, United Kingdom; 6 Oxford Health NHS Foundation Trust, Warneford Hospital, Oxford, United Kingdom; 7 Mansfield Centre for Innovation - MR Facility, MRC Laboratory of Medical Sciences, Hammersmith Hospital, London, United Kingdom; 8 Division of Psychology and Mental Health, Faculty of Biology, Medicine, and Health, University of Manchester, Manchester, United Kingdom; 9 Department of Neuroimaging, King’s College London, London, United Kingdom; 10 Institute of Neuroscience, Newcastle University, Newcastle upon Tyne, United Kingdom; 11 Queensland Brain Institute, The University of Queensland, Queensland, Australia; 12 Department of Radiology, Royal Melbourne Hospital, University of Melbourne, Victoria, Australia; 13 Department of Information Engineering, University of Padua, Padova, Italy; 14 Sumitomo Pharma America, Inc., Marlborough, Massachusetts, USA

## Abstract

**Objective:**

Neuroimaging studies have independently associated schizophrenia with low iron and elevated dopamine synthesis. While preclinical research demonstrates that midbrain iron deficiency leads to striatal hyperdopaminergia, this relationship has not been studied in schizophrenia. We therefore examined case-control differences in tissue magnetic susceptibility, a marker of brain iron, and correlated this with striatal dopamine synthesis capacity.

**Methods:**

Magnetic susceptibility in the substantia nigra and ventral tegmental area (SN-VTA) was measured using quantitative susceptibility mapping (QSM) MRI in 159 participants (controls N=80; early course schizophrenia N=79 including antipsychotic naïve/free patients). As magnetic susceptibility is increased by neuromelanin and reduced by myelin, neuromelanin-sensitive MRI (NM-MRI) and diffusion tensor imaging (DTI) were employed to investigate their influence on the QSM findings in 113 participants (controls N=38; schizophrenia N=61). Dopamine synthesis capacity (Ki^cer^) was then assessed with [18F]-DOPA PET in 40 people from the schizophrenia group to test if low SN-VTA magnetic susceptibility was related to high striatal Ki^cer^.

**Results:**

SN-VTA magnetic susceptibility was lower in patients with schizophrenia than controls (d=-0.66; 95% CI=-0.98, -0.34), which remained significant when controlling for mean diffusivity (a DTI measure inversely correlating with myelin concentration) and NM-MRI contrast-to-noise ratios (p<0.001). SN-VTA magnetic susceptibility inversely correlated with striatal Ki^cer^, independent of mean diffusivity and NM-MRI contrast-to-noise ratios (r=-0.44, p=0.005). In both analyses, the strongest effects were observed in the ventral SN-VTA.

**Conclusions:**

These findings suggest that lower SN-VTA non-neuromelanin-bound iron contributes to striatal hyperdopaminergia in schizophrenia and supports further investigation into the role of low iron in schizophrenia pathophysiology and its potential as a treatment target.

## Introduction

Central to the dopamine hypothesis of schizophrenia is the idea that increased presynaptic striatal dopamine synthesis underlies disease pathophysiology (1). Dopamine neurons projecting to the striatum via the mesostriatal pathway originate in the substantia nigra and ventral tegmental area (SN-VTA) (1). In these neurons, cytosolic dopamine is metabolized into neuromelanin, which is a marker of long-term dopaminergic activity (2, 3). When bound to iron, neuromelanin is detectable using neuromelanin-sensitive MRI (NM-MRI) (4). The discoveries that SN-VTA NM-MRI contrast-to-noise ratios (NM-CNRs) are elevated in schizophrenia relative to controls (5, 6), and that NM-CNR correlates with striatal dopamine synthesis (6) and release (7), indicate that striatal dopamine dysfunction may originate in the SN-VTA.

Since greater iron binding in neuromelanin enhances NM-MRI signal (4), elevated NM-CNRs in schizophrenia could reflect higher levels of iron, neuromelanin, or both. Iron is an essential cofactor for both dopamine and neuromelanin synthesis (2, 8); therefore, increased iron levels in schizophrenia might account for the elevated NM-CNR by directly promoting neuromelanin production and indirectly amplifying it through increased dopamine availability. However, evidence links low iron levels to schizophrenia. Specifically, low blood iron has been reported in individuals with schizophrenia (9) and in pregnant women whose children later develop the condition (10). Moreover, iron-sensitive MRI studies in early schizophrenia have shown lower iron signal in the putamen (11, 12) and SN-VTA (12, 13) relative to controls.

A link between low iron and high neuromelanin in schizophrenia may be explained by research indicating that iron deficiency leads to hyperdopaminergia (14–17). For instance, rodents that were fed an iron-deficient diet exhibited reduced brain iron levels (14–17) and increased striatal synaptic dopamine concentrations (14–17), which normalize when iron is replenished in the SN-VTA (15), and increased dopamine synthesis (14, 16). This suggests low iron could drive the elevated dopamine synthesis capacity and neuromelanin levels observed in schizophrenia. However, the relationship between these factors has not previously been investigated in schizophrenia.

We recently reported that SN-VTA NM-CNR was higher in schizophrenia than in controls and that this correlated with striatal dopamine synthesis capacity (Ki^cer^) (6). In the current paper, we further explore SN-VTA function in these participants using quantitative susceptibility mapping (QSM) to assess SN-VTA magnetic susceptibility (shortened to susceptibility hereafter), which correlates with subcortical iron concentration (18). As susceptibility is reduced by myelin (18), we utilized diffusion tensor imaging (DTI) to calculate tissue mean diffusivity, which inversely correlates with myelin concentration (19). In our first analysis, we hypothesized that SN-VTA susceptibility would be lower in people with schizophrenia than matched controls when adjusting for SN-VTA mean diffusivity and NM-CNR, linking schizophrenia to low non-neuromelanin-bound iron. In our second analysis, we used 6-[18F]fluoro-dihydroxyphenyl-l-alanine ([18F]-DOPA) positron emission tomography (PET) to measure Ki^cer^ in a subsample of the schizophrenia group. We predicted an inverse correlation between SN-VTA susceptibility and striatal Ki^cer^, when controlling for SN-VTA mean diffusivity and NM-CNR, thereby associating low non-neuromelanin-bound iron to elevated striatal dopamine function in schizophrenia for the first time.

## Methods

### Participants

We obtained recruitment approval from the London-Dulwich Research Ethics Committee for healthy controls and the Office for Research Ethics Committees Northern Ireland for patients with schizophrenia. This paper only presents baseline data from an ongoing clinical study (NCT04038957). Written informed consent was obtained from all volunteers aged 18-45 years, to examine patients with early illness.

Patients with schizophrenia were recruited from community mental health teams in London, UK. Schizophrenia diagnosis was confirmed by a study psychiatrist using the Structured Clinical Interview for DSM-5 (SCID-5) (20) and a review of the clinical notes. Exclusion criteria included other psychiatric disorders, substance use disorder (excluding nicotine), use of more than one antipsychotic, non-antipsychotic psychotropics, and current or past clozapine use.

We recruited healthy controls via advertisement from London, UK. They underwent an interview with a study psychiatrist and were excluded if they had a history of mental illness, substance use disorder (other than nicotine), psychotropic medication use, or family history of schizophrenia.

As recent use of stimulants could affect the dopaminergic system (21), participants completed a urine drug screen (UDS) immediately before the MRI and were excluded if this was positive for any substance other than delta-9-tetrahydrocannabinol (THC), given its prolonged detectability (22). Smoking status was categorized as current smoker, former smoker, or never smoked. Additional exclusion criteria are detailed in the Supplementary Methods.

### Clinical Assessments

Clinico-demographic details were collected for all volunteers. Patients underwent the Positive and Negative Syndrome Scale (PANSS) (23), Brief Negative Symptoms Scale (BNSS) (24), and Clinical Global Impression-Severity Scale (CGI-S) (25). The number of patients with a total PANSS score ≥75, indicating moderate psychosis (26), was calculated. Antipsychotic doses were converted to chlorpromazine daily equivalents (27).

### MRI Acquisition and Processing

Table 1 outlines the MRI sequence acquisition parameters. Voxel NM-MRI signal was converted into an NM-CNR, a reliable marker of neuromelanin concentration (7). Full processing and quality control steps for MRI data are in the Supplementary Methods. MRI images were normalized to the Montreal Neurological Imaging (MNI) space. The SN-VTA mask (Supplementary Figures S1 & S2; accessible from: https://github.com/lukevano/KCL_Neuromelanin-MRI) was moved from MNI to the participant’s space for each of their MRI images using Advanced Normalization Tools (ANTs) 2.4.0 (28). A striatal atlas (29) was also moved into each participant’s QSM space. The mean value for voxels in each mask was used for the region of interest analysis. Susceptibility was recorded in parts per billion (ppb). Normalized QSM images were spatially smoothed with a 1-mm sigma Gaussian kernel before voxelwise analysis.

### PET acquisition and Processing

Our standard procedure was used to acquire and process the PET-MRI data (30) acquired on a SIGNA General Electric 3T PET/MR scanner. Carbidopa (150 mg) and entacapone (400 mg) were orally administered 1 hour before the scan to enhance the signal-to-noise ratio (30). The maximum activity of administered [18F]-DOPA was 150MBq. Dopamine synthesis capacity was measured using the Patlak-Gjedde method for calculating the Ki^cer^ (min^−1^) of [18F]-DOPA uptake relative to the cerebellum as the reference region (30). Ki^cer^ values were obtained for the whole striatum, striatal subregions (limbic, associative, and sensorimotor), and the SN-VTA in each participant’s PET space. Participant parametric Ki^cer^ maps were generated, transformed into MNI space, and smoothed by a 1-mm sigma Gaussian kernel for exploratory voxelwise analysis. Further PET processing details are in the Supplementary Methods.

### Statistical analysis

Data analysis was conducted using in-house Python scripts. We controlled for the following potential clinical confounders in our analyses: age, sex, current or past smoking history, and THC-positive UDS. In additional analyses, we also controlled for SN-VTA NM-CNR and mean diffusivity. Independent sample t-tests and Cohen’s d were used to assess group differences in continuous variables, and chi-squared tests for categorical variables. Pearson’s r was used to assess correlation. For exploratory analyses, the Benjamini-Hochberg method was applied to control the false discovery rate (FDR) at p<0.05.

### Case-control SN-VTA susceptibility differences

Robust linear regression modeled the relationship between case-control status, clinical confounders, and SN-VTA susceptibility. SN-VTA NM-CNR and mean diffusivity were added in an additional model. Antipsychotic-free patients were compared to those on antipsychotics when controlling for confounders. SN-VTA susceptibility was correlated with clinical variables and antipsychotic dose. The statistical significance of these correlations was tested using robust linear regression models adjusted for confounders, with FDR correction applied. A sensitivity analysis repeating this in antipsychotic-free patients was also performed in case treatment influenced our results. Additionally, SN-VTA susceptibility was correlated with age across all participants, and with NM-CNR for the patient and control groups. The significance of differences between patient and control correlations was evaluated using Fisher’s r-to-z transformation, implemented with the R package cocor (31).

We conducted a moderation analysis to assess whether age influenced the relationship between SN-VTA susceptibility and NM-CNR. A robust linear regression model predicted NM-CNR using SN-VTA susceptibility, age, and their interaction, with predictors mean centered to zero to address multicollinearity.

Voxelwise analysis used robust linear regression to predict SN-VTA susceptibility based on case-control status and clinical confounders. Voxel data with susceptibility beyond the 1st and 99th percentiles of values in the whole sample (-38 and 234 ppb, respectively) were censored. Threshold-free cluster enhancement (TFCE) was applied for cluster inference combined with permutation testing (10,000 iterations) (32). FDR correction was used to determine significant clusters (p<0.05). We used the same method to predict PANSS positive score from voxel susceptibility in the total patient population and the antipsychotic-free cohort. Spatial extent voxelwise analysis was also performed to identify voxels of significant case-control difference.

### Relationship between striatal Ki^cer^ with susceptibility in schizophrenia

In patients with PET scans, we correlated SN-VTA susceptibility with striatal Ki^cer^, including a moderation analysis to assess the influence of either age or NM-CNR on this association, following the method outlined above. Robust linear regression also modeled this relationship, when controlling for clinical confounders, NM-CNR, and mean diffusivity. Exploratory analyses included FDR-controlled correlations between SN-VTA susceptibility with Ki^cer^ in striatal subdivisions and SN-VTA and correlating striatal susceptibility with striatal Ki^cer^. Differences between subregion correlations were tested using the Steiger method (31).

We used partial least squares regression (PLSR) to examine the voxelwise relationship between SN-VTA susceptibility and striatal K_i_^cer^, as PLSR handles high-dimensional data with multicollinearity by generating latent variables (“scores”) that maximize the correlation between predictors (SN-VTA susceptibility) and responses (striatal K_i_^cer^) (6, 33, 34). To avoid overfitting, we identified only one pair of latent variables. The effects of clinical confounders were regressed out of predictor and response variables before constructing the model using the nonlinear iterative partial least squares algorithm in the Python package scikit-learn (35).

We assessed model significance using randomized 10-fold cross-validation to generate out-of-sample scores. This was repeated 100 times and the average Pearson’s r of these out-of-sample scores was compared to a null distribution from 10,000 subject-level permuted models to determine statistical significance. Since the sign of scores produced by PLSR is arbitrary, the signs of the striatal K_i_^cer^ scores were reversed if score correlation directionality did not align with the correlation between SN-VTA susceptibility and striatal K_i_^cer^. Voxel contributions were assessed using z-scores, derived from variable importance in projection (VIP) scores, with error estimated from 1,000 bootstrapped runs (resampling with replacement of the striatal K_i_^cer^ voxels) (34). Z-scores were visualized on 2D and 3D maps over the QSM template.

## Results

### Participant details

We used QSM in 171 participants (controls N=86; early course schizophrenia N=85, including N=20 antipsychotic-free). After excluding 13 scans due to excessive movement (controls N=3; schizophrenia N=4) or artifacts (controls N=3; schizophrenia N=2), 80 controls and 79 individuals with schizophrenia had analyzable QSM data. As the NM-MRI and DTI protocols were added later, only 141 participants had NM-MRI data (controls N=73; schizophrenia N=68), and 99 participants completed both scans (controls N=38; schizophrenia N=61). PET scans were analyzed in 40 participants, after excluding 2 for motion. Thirty-three schizophrenia patients had complete neuroimaging data. Baseline clinic-demographic data (Table 2) showed no significant group differences in age, sex, ethnicity, or THC-positive status; however, smoking status differed significantly (p=0.007), with more current smokers in the schizophrenia (37%) than control (15%) group.

### Case-control differences in SN-VTA susceptibility

**Figure 1:**
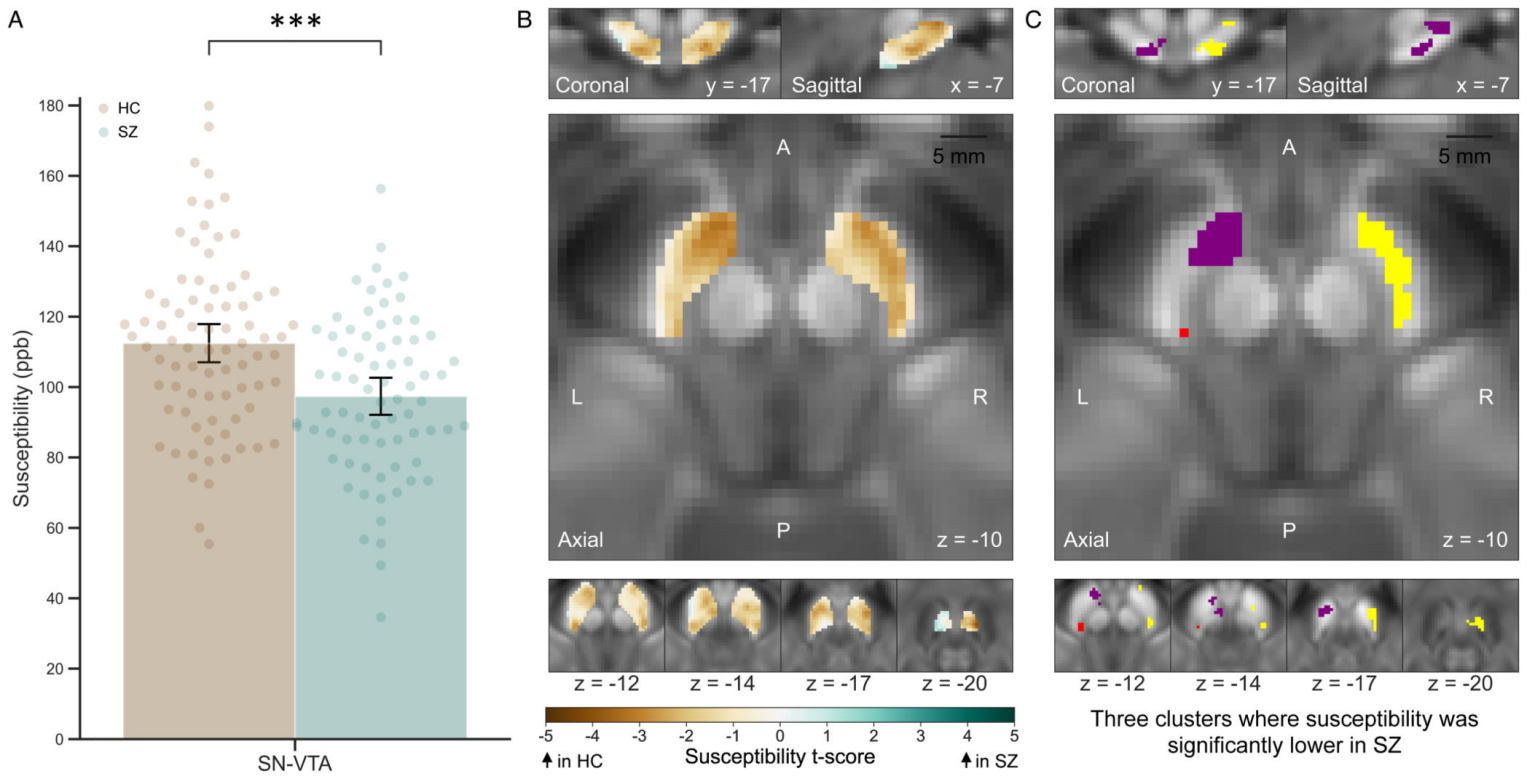
Substantia nigra and ventral tegmental area (SN-VTA) quantitative susceptibility mapping (QSM) case-control analysis ^a^ ^**a**^ Panel A is a bar chart showing mean SN-VTA susceptibility, calculated by QSM, for the groups (95% confidence intervals) with values also plotted for each participant. Healthy control values are displayed in brown and data for patients with schizophrenia are in green. Susceptibility was significantly lower for participants with schizophrenia relative to the control group (d=-0.66; 95% CI=-0.98, -0.34). This remained significant when controlling for potential clinical confounders (p<0.001). Panel B shows the SN-VTA t-score map. The colorbar refers to the t-score, where brown indicates lower susceptibility in schizophrenia relative to controls and green lower susceptibility in controls. Panel C displays the 3 clusters (327 voxels out of the 1790 SN-VTA voxels) where schizophrenia was associated with significantly lower susceptibility (threshold-free cluster enhancement, Benjamini-Hochberg corrected p<0.05). The peak t-score of each cluster was in the right ventrolateral SN-VTA (x=7, y=-22, z=−20; t=-3.98; n voxels=187; yellow), left ventral SN-VTA (x=-7, y=-11, z=−10; t=-3.28; n voxels=114; purple), and left lateral SN-VTA (x=-11, y=-23, z=−13; t=-2.82; n voxels=26; red). ***=p<0.001. ppb=parts per billion.

SN-VTA susceptibility was significantly lower in patients with schizophrenia compared to controls (d=-0.66; 95% CI=-0.98, -0.34; Figure 1A) and remained significant after controlling for clinical confounders (t=-3.82, p<0.001; Table 3). SN-VTA susceptibility was positively associated with age (t=2.38, p=0.017; r=0.19, p=0.018; Supplementary Figure S3), with no significant relationships observed with sex (p=0.387), THC-positive status (p=0.859), current (p=0.27) or past smoking (p=0.737). When controlling for SN-VTA mean diffusivity and NM-CNR, susceptibility remained significantly lower in schizophrenia (t=-3.38, p<0.001). While SN-VTA mean diffusivity did not significantly affect susceptibility (p=0.394), NM-CNR had an inverse relationship (t=-2.38, p=0.017). SN-VTA susceptibility correlated positively with NM-CNR in controls (r=0.25, p=0.032; Supplementary Figure S4A) but not in the schizophrenia group (r=-0.19, p=0.111; Supplementary Figure S4B). These correlation coefficients were significantly different (z=2.6, p=0.009). Age did not moderate these relationships in the controls (p=0.107) or patients (p=0.831).

SN-VTA susceptibility was not significantly different between patients taking an antipsychotic and those who were not (t=-1.13, p=0.262; Supplementary Figure S5), and did not correlate significantly with any clinical measure or chlorpromazine daily equivalent antipsychotic dose (Supplementary Figure S6A). No significant correlation between SN-VTA susceptibility and clinical measures was found in the antipsychotic-free patient group (Supplementary Figure S6B).

Voxelwise analysis revealed 3 clusters (327/1790 SN-VTA voxels) with significantly lower susceptibility in schizophrenia, predominantly in the ventral and lateral SN-VTA (Figure 1B-C). The spatial extent method identified the same relationship in voxels overlapping these clusters (Supplementary Figure S7). No significant clusters or voxels were identified linking schizophrenia with higher susceptibility. Additionally, one significant cluster in the right medial SN-VTA showed a negative correlation with PANSS positive score (Supplementary Figure S8). No clusters were identified showing a positive correlation in this group, or any relationship in the antipsychotic-free patients.

### Relationship between striatal Ki^cer^ and SN-VTA susceptibility in schizophrenia

The results of these analyses are shown in Supplementary Figure S9 and Table S2. SN-VTA susceptibility was negatively correlated with striatal Ki^cer^ (r=-0.44, p=0.005), a relationship that remained significant after adjusting for mean diffusivity, NM-CNR, and confounders (t=-2.88, p=0.004). This correlation was not modulated by age (p=0.931) or NM-CNR (p=0.568). Striatal Ki^cer^ was negatively associated with current smoking status (t=-2.81, p=0.005), age (t=-3.26, p<0.001), and male sex (t=-4.74, p<0.001); positively associated with THC-positive status (t=2.66, p=0.008) and NM-CNR (t=2.27, p=0.023); and not significantly associated with SN-VTA mean diffusivity (p=0.328) or past smoking (p=0.27).

Exploratory analysis revealed significant inverse correlations (FDR corrected) between SN-VTA susceptibility and striatal Ki^cer^ in the associative (r=-0.45, p=0.015) and sensorimotor (r=-0.43, p=0.015) subregions, but not the limbic subregion (r=-0.14, p=0.402). Associative and sensorimotor correlations were similar (p=0.7352), but both significantly differed from the limbic correlation (p<0.001 and p=0.031, respectively). Significant correlations were not identified between striatal Ki^cer^ and striatal susceptibility (r=-0.19, p=0.393), or SN-VTA susceptibility with SN-VTA Ki^cer^ (r=-0.15, p=0.402).

**Figure 2:**
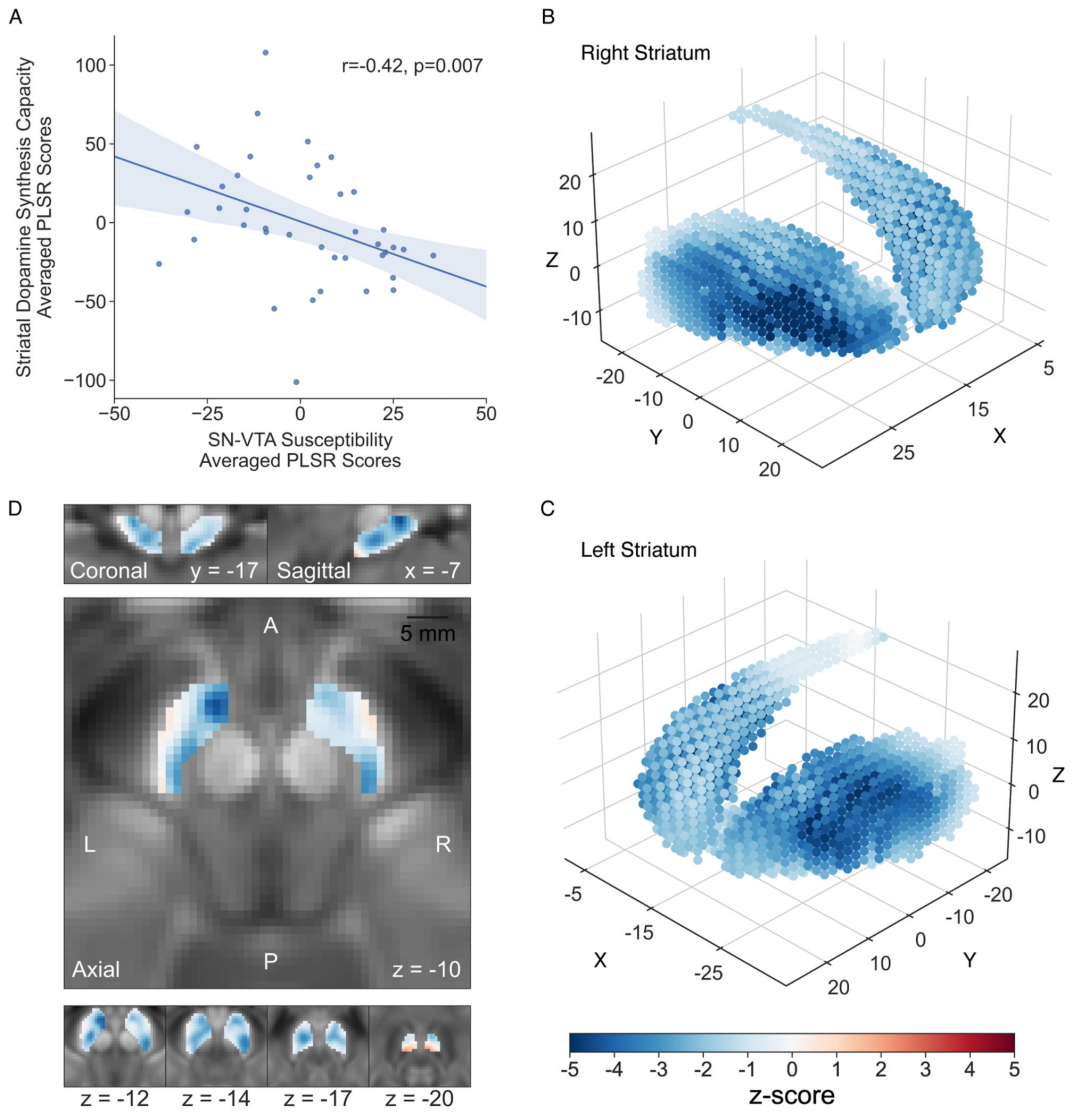
Association between the voxelwise pattern of striatal dopamine synthesis capacity (K_i_^cer^) and substantia nigra and ventral tegmental area (SN-VTA) susceptibility ^a^ ^**a**^ Panel A shows the correlation between the average partial least squares regression (PLSR) generated out-of-sample scores assessing the voxelwise relationship between striatal K_i_^cer^ and SN-VTA susceptibility. In panels B and C, z-score maps show the strength of relationship between SN-VTA susceptibility and each striatal voxel K_i_^cer^. Positive z-scores (red) indicated a direct relationship with SN-VTA susceptibility, while negative z-scores (blue) demonstrated an inverse relationship. The peak striatal z-score was in the right dorsal striatum (x=28, y=-4, z=−8; z-score=-4.46). Panel D displays the SN-VTA z-score map showing the strength of the voxel relationship with striatal K_i_^cer^. The peak z-score was in the left ventral SN-VTA (x=-5, y=-12, z=−11; z-score=-4.56).

The correlation between the average out-of-sample generated PLSR scores was significant on permutation testing (r=-0.42, p=0.007; Figure 2A), indicating a relationship between the pattern of SN-VTA susceptibility and striatal K_i_^cer^. Z-score maps showed that 1680/1790 SN-VTA and 2908/2967 striatal voxels had negative values, highlighting the inverse correlation between these variables (Figure 2B-D). Peak z-scores were in the right dorsal striatum and left ventral SN-VTA.

## Discussion

We extend previous findings that schizophrenia is associated with lower SN-VTA iron-sensitive MRI signal than controls (12, 13) by showing this in a larger sample and identifying, for the first time, clusters in the ventral and lateral SN-VTA where this difference is significant. This effect remained when controlling for SN-VTA mean diffusivity, a measure inversely correlating with myelin concentration (19), indicating that lower SN-VTA susceptibility in schizophrenia is driven by lower iron rather than higher myelin levels. This effect persisted after accounting for SN-VTA NM-CNR, which we previously showed was higher in this schizophrenia group relative to the controls (6). Our findings indicate that the lower SN-VTA iron-sensitive MRI signal in schizophrenia is due to less non-neuromelanin-bound iron.

We identified a negative correlation between SN-VTA susceptibility and striatal Ki^cer^, which was strongest in the dorsal striatum—the main locus of hyperdopaminergia in schizophrenia (1). This remained significant when controlling for NM-CNR; meaning our results link, for the first time, lower SN-VTA non-neuromelanin-bound iron in schizophrenia to higher striatal dopamine synthesis capacity, consistent with our hypothesis that reduced iron levels could underlie hyperdopaminergia in schizophrenia.

### Methodological considerations

A strength of our study is the sample size, which is the largest QSM schizophrenia study to date, minimizing the risk of type II error. Additionally, most patients were experiencing at least moderately severe psychotic symptoms, adding clinical relevance. While basal ganglia susceptibility correlates with postmortem iron levels (18), this correlation can be affected by the postprocessing method. We used a postprocessing pipeline validated to measure tissue iron in resected brain samples (36).

Medication use may be a confounder. However, a previous QSM study in first-episode psychosis found no effect of antipsychotic dose or treatment duration on basal ganglia susceptibility (11). Longitudinal evidence also shows that antipsychotic treatment does not significantly alter striatal K_i_^cer^ in first-episode psychosis (37). This, alongside our findings showing no association between medication status or chlorpromazine equivalent dose with susceptibility, indicates that medication exposure is unlikely to confound our results.

As with previous studies (13, 38), we did not identify a relationship between whole SN-VTA susceptibility and clinical measures. However, our voxelwise analysis revealed a cluster in the right medial SN-VTA where susceptibility negatively correlated with PANSS positive symptom severity score. Therefore, iron loss in this part of the SN-VTA may be associated with psychotic symptoms.

A limitation is that we did not control for alcohol use. Although we excluded participants meeting the criteria for alcohol use disorder, even moderate alcohol consumption (≥6 units per week) has been linked to increased subcortical susceptibility (39). Given that higher alcohol use is generally reported in schizophrenia (40), this is unlikely to explain the lower susceptibility we observed in this group.

### The origin of lower SN-VTA susceptibility in schizophrenia

QSM measures overall voxel susceptibility (18), summing components with positive (e.g., iron) and negative (e.g., myelin) susceptibilities. Our finding that SN-VTA susceptibility remained significantly lower in schizophrenia after controlling for mean diffusivity, which inversely correlates with myelin and tissue density (19), suggests that lower iron rather than altered myelin or tissue density underlies this effect. This is supported by a case-control study using MRI techniques with specific sensitivity for iron and myelin (12), which associated schizophrenia with lower SN-VTA iron-sensitive signal without group differences in the myelin-sensitive marker. Moreover, postmortem studies have linked schizophrenia to myelin loss (41), and iron deficiency is known to impair myelin synthesis (8). Thus, lower susceptibility in schizophrenia likely reflects lower iron levels, without myelin changes or despite myelin loss.

Approximately 20% of iron in the SN-VTA is bound to neuromelanin, 75% is stored in ferritin, and 5% resides in the labile pool (42), which contains redox-active iron essential for biological processes (8). Susceptibility reflects iron contributions from all these sources (42), whereas NM-MRI only measures neuromelanin-bound iron (4). Our finding that SN-VTA susceptibility did not correlate with NM-CNR in schizophrenia, despite a positive correlation in controls, along with higher NM-CNR (6) and lower susceptibility in the patient group, suggests that neuromelanin-bound iron is elevated in schizophrenia (6), while non-neuromelanin-bound iron is reduced.

SN-VTA neuromelanin is detectable only in dopamine neurons, which contain minimal ferritin (2, 43)—the primary brain iron storage protein (2). In iron-deficient states, ferritin is destroyed to release iron into the labile iron pool (2). Since neuromelanin chelates iron and is not broken down under physiological conditions (3), it effectively sequesters iron from the labile iron pool. Therefore, our results suggest that in schizophrenia, there is less bioavailable iron in the SN-VTA; increasing the risk of local iron deficiency.

### The relationship between SN-VTA iron and striatal dopamine synthesis in schizophrenia

We demonstrate that SN-VTA—but not striatal—susceptibility is inversely correlated with striatal K_i_^cer^ in schizophrenia. This relationship remained significant after controlling for NM-CNR and mean diffusivity, indicating that reduced SN-VTA non-neuromelanin-bound iron is associated with elevated striatal K_i_^cer^. K_i_^cer^ indexes [18F]-DOPA uptake into dopamine neurons, conversion to dopamine by aromatic amino-acid decarboxylase, and subsequent vesicular uptake via vesicular monoamine transporter 2 (VMAT2) expressed at presynaptic terminals (30). Therefore, our findings could be explained by low iron either enhancing dopamine synthesis or increasing presynaptic vesicular uptake and storage of dopamine.

A study using [11C]dihydrotetrabenazine PET, which indexes VMAT2 availability, found a direct correlation between iron-sensitive MRI signal and [11C]dihydrotetrabenazine uptake in the limbic but not dorsal striatum of young adults (44). Since our SN-VTA susceptibility effect was observed within the dorsal striatum, VMAT2 variation is unlikely to explain our findings, but warrants testing in future studies.

Human studies examining the link between iron and dopamine function are limited, but extensive preclinical research has explored this relationship. In rodent models, reversible brain iron deficiency is induced by an iron-deficient diet (14–17) and leads to increasing synaptic striatal dopamine levels (14–17). Infusing iron into the SN-VTA—but not the striatum—ameliorated this striatal dopamine dysfunction (15), in line with our finding connecting SN-VTA iron with striatal dopamine function.

Iron is an essential cofactor for tyrosine hydroxylase (TH), the enzyme catalyzing the rate-limiting step in dopamine synthesis. Despite this, iron-deficient rodents exhibit increased total and active (phosphorylated) TH in the striatum (16) and SN-VTA (14). Similarly, applying the iron chelator desferrioxamine, which reduces free iron, to catecholaminergic cells also increased TH levels (14). Therefore, low SN-VTA non-neuromelanin-bound iron could upregulate dopamine synthesis in schizophrenia—however, further investigation is needed to confirm this hypothesis and elucidate the underlying mechanisms.

Our prior finding of a positive correlation between striatal K_i_^cer^ and SN-VTA NM-CNR in the same cohort (6) suggests that increased dopamine synthesis may drive elevated neuromelanin synthesis in schizophrenia. As discussed above, the observed negative correlation between SN-VTA susceptibility and striatal K_i_^cer^ may indicate that reduced non-neuromelanin-bound iron contributes to striatal hyperdopaminergia, ultimately leading to higher neuromelanin levels in schizophrenia. Future longitudinal studies in early-stage schizophrenia are essential to further investigate this relationship and its progression over time.

### Future directions

Several lines of evidence implicate low iron levels in the pathoetiology of schizophrenia. Epidemiological studies link famine to an increased schizophrenia risk, with iron deficiency highlighted as a key micronutrient of interest (45). Maternal anemia has been shown to nearly quadruple the risk of schizophrenia spectrum disorder in offspring (10). Moreover, low blood iron levels are associated with schizophrenia (9) and more severe symptoms (46). Iron-sensitive MRI studies show reduced subcortical iron in early schizophrenia (11–13), consistent with our findings. Since blood iron correlates with subcortical susceptibility (39), dietary iron deficiency may contribute to reduced brain iron. However, studies confirming this relationship in schizophrenia are required before considering iron supplementation as a treatment.

In contrast to early-stage disease, chronic schizophrenia is associated with greater iron-sensitive signal in the thalamus (47) and putamen (38). Furthermore, postmortem analysis of the cortex has linked schizophrenia to greater iron and lower ferritin (48). Future exploration of subcortical iron distribution compartments—such as ferritin, neuromelanin, and the labile iron pool—would be useful to understand if there are changes during the course of schizophrenia.

Our voxelwise analysis identified the ventral and lateral SN-VTA as regions of significantly lower susceptibility in schizophrenia. Susceptibility in the ventral SN-VTA showed the strongest inverse relationship with dopamine synthesis capacity in the dorsal striatum. The ventral SN-VTA has also been highlighted as a region of interest in recent NM-MRI studies, which have shown that NM-CNR here correlated with psychotic symptoms (7, 49); a diagnosis of treatment-responsive schizophrenia (50); and both dopamine release (7) and synthesis capacity (6) in the associative striatum. Our findings further support the ventral SN-VTA as a critical area where dysfunction may contribute to the pathophysiology of schizophrenia.

## Conclusion

Using QSM, DTI, and NM-MRI, we provide evidence that lower SN-VTA magnetic susceptibility in schizophrenia relative to controls is due to reduced non-neuromelanin-bound iron levels. We identified an inverse relationship between SN-VTA susceptibility and striatal dopamine synthesis capacity in schizophrenia. These relationships appeared most significant in the ventral SN-VTA. Our findings highlight low brain iron in schizophrenia as a potential biomarker and treatment target.

## Supplementary Material

Supplement

## Figures and Tables

**Table 1:** MRI acquisition parameters ^a^

	T1-weighted	QSM	DTI	NM-MRI
Slices	176	144	27	12
Inplane resolution, mm	1 x 1	1 x 1	1.7 x 1.7	0.8 x 0.8
Slice thickness, mm	1	1	4	2
Echo time, ms	2.91	5.84 (then 8 subsequent echoes with 4.79ms spacing)	69	2.85
Repetition time, ms	2300	50	3200	359
Flip angle, °	9	15	-	40
b-value, s/mm^2^	-	-	64 directions b=0 (11 avg) b=1000 (2 avg)	-
MT Pulse flip angle, °	-	-	-	500
MT Pulse offset, Hz	-	-	-	1200
Bandwidth, Hz/pixel	140	310	1954	320
Number of averages	1	1	1	5
Acceleration, GRAPPA	2	3	2	none
Acquisition time, min:s	5:35	8:05	7:36	5:50

a MRI was performed on a 3T MAGNETOM Prisma (Siemens Healthineers, Erlangen, Germany) using a 64-channel head coil. The following sequences were used: Magnetization prepared rapid gradient echo (MPRAGE; T1-weighted), a multi-echo 3D gradient recall echo sequence to calculate the quantitative susceptibility mapping (QSM) images, a single-shot echo-planar diffusion tensor imaging (DTI) acquisition followed by a reversed-phase encoding direction B0 volume for distortion correction, and an optimized 2D gradient echo neuromelanin-sensitive MRI (NM-MRI) sequence. mm=millimeters; ms=milliseconds; °=angle; MT=magnetization transfer; Hz=Hertz; GRAPPA=GeneRalized Autocalibrating Partial Parallel Acquisition; min=minutes; s=seconds; avg=average.

**Table 2:** Baseline clinic-demographic characteristics and experimental values for participants with analyzable data ^a^

Characteristic	Healthy Controls (N=80)		Schizophrenia (N=79)		p
	N	%	N	%	
Male	56	70	56	71	1
Ethnicity					0.775
Asian	11	14	10	13	
Black	36	45	41	52	
White	28	35	24	30	
Mixed	5	6	4	5	
Smoking Status					0.007
Current Smoker	12	15	29	37	
Past Smoker	14	18	9	11	
Never Smoked	54	67	41	52	
THC-positive UDS	19	24	20	25	0.964
PANSS Total ≥ 75			46	58	
	Mean	SD	Mean	SD	p
Age	31.9	6.4	31.2	7	0.5
Neuroimaging Measures					
N with NM-MRI	73		68		
N with NM-MRI and DTI	38		61		
N with PET	0	0	40		
N with NM-MRI, DTI, & PET			33		
Days Between MRI and PET			11	11	
Injected Activity (MBq)			141.7	5.1	
Clinical Measures					
PANSS Total			73	14.1	
PANSS Positive			17	5.4	
PANSS Negative			20.3	5.7	
PANSS General			36	7.2	
BNSS			30.6	14.6	
CGI-S			4	0.9	
Current Medication					
Chlorpromazine Daily Equivalent Doses (mg)			233.7	151.6	
Aripiprazole			21		
Olanzapine			19		
Risperidone			7		
Paliperidone			7		
Lurasidone			3		
Flupentixol			2		
Unmedicated			20		
			Median	IQR	
Duration of Illness (Years)			2.7	2.5	

a N=number of participants; p=p-value; SD=standard deviation THC=delta-9-tetrahydrocannabinol; UDS=urine drug screen; DTI=diffusion tensor imaging; QC=quality control; PET=Positron emission tomography; NM-MRI=Neuromelanin-sensitive MRI; MBq=megabecquerel; mg=milligram; PANSS=Positive and Negative Syndrome Scale; BNSS=Brief Negative Symptoms Scale; CGI-S=Clinical Global Impression-Severity Scale; IQR=interquartile range.

**Table 3:** Results from the robust linear model built to predict substantia nigra and ventral tegmental area (SN-VTA) magnetic susceptibility (ppb) with case-control status and potential confounders (controls N=80; schizophrenia N=79) ^a^

Variable	Coefficient	Standard Error	t-score	p-value	[95% CI]	
(Intercept)	93.91	9.66	9.72	<0.001	74.98	112.84
Schizophrenia Group Status	-14.24	3.73	-3.82	<0.001	-21.54	-6.93
Current Smoker	5.32	4.81	1.10	0.27	-4.12	14.75
Past Smoker	-1.87	5.56	-0.34	0.737	-12.77	9.03
THC-positive UDS	0.83	4.66	0.18	0.859	-8.30	9.96
Male Sex	-3.48	4.02	-0.86	0.387	-11.37	4.41
Age	0.66	0.28	2.38	0.017	0.12	1.20

a ppb=parts per billion; CI=confidence interval; THC=Delta-9-tetrahydrocannabinol; UDS=urine drug screen.

## Plain Language Summary

While schizophrenia has been associated with low blood iron levels and high striatal dopamine synthesis, a link between these factors has not previously been explored. Using brain imaging, this study provides evidence of an inverse relationship between brain iron and dopamine synthesis. This suggests that treating low brain iron may be of benefit in schizophrenia.
