# Supplementary material for: Reduced Brain Iron is Associated with Striatal Hyperdopaminergia in Schizophrenia and Unrelated to Neuromelanin and Myelin Imaging Measures: A Quantitative Susceptibility Mapping MRI and PET Study
Source: Am J Psychiatry. Author manuscript; Available in PMC 2025 Oct 4. (PMC7618194; doi:10.1176/appi.ajp.20240512)
Supplement: Supplement [file EMS208752-supplement-Supplement.pdf]

Supplementary Figures

Figure S1. Substantia nigra and ventral tegmental area (SN-VTA) mask on the quantitative susceptibility mapping (QSM) template [a]

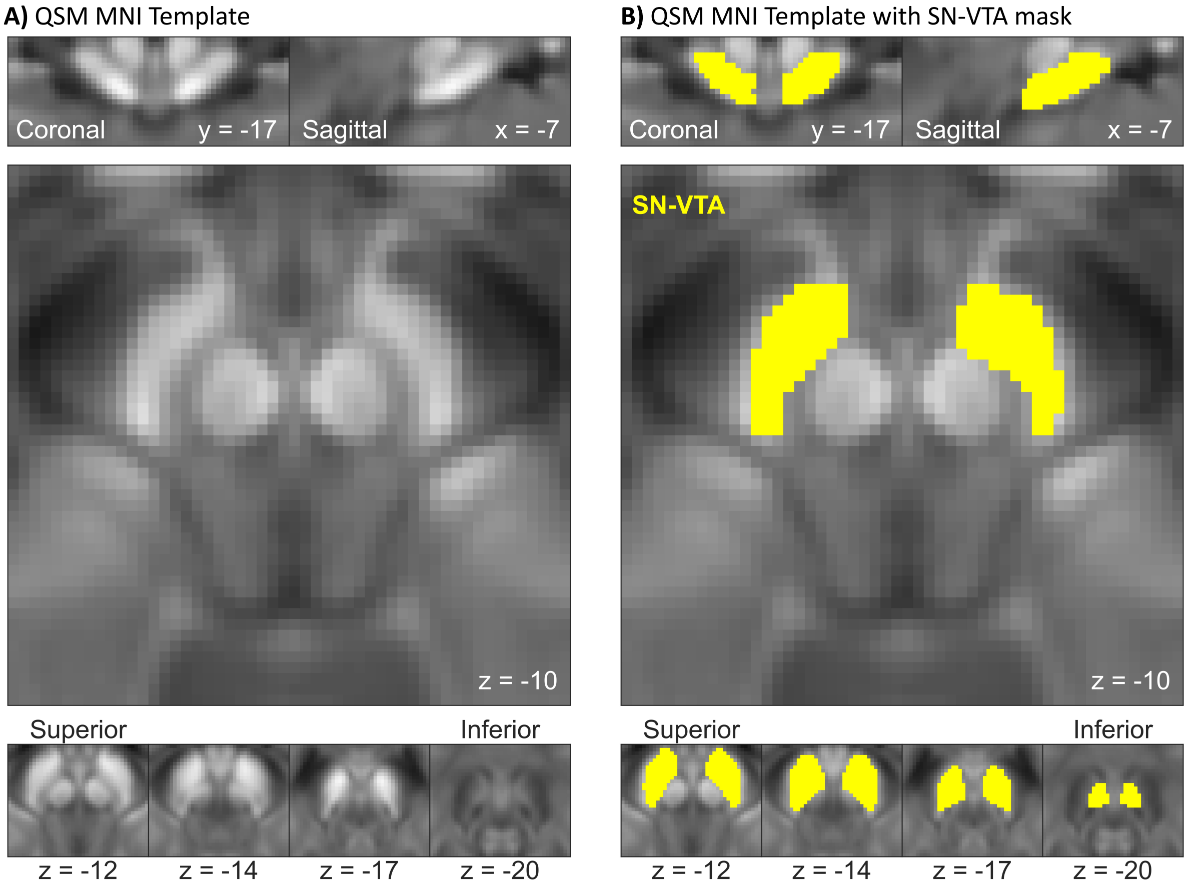

[a] Panel a displays the study QSM template generated by averaging QSM images normalized to the Montreal Neurological Imaging (MNI) space. Panel b shows the SN-VTA mask (yellow) on this template.

Figure S2. Substantia nigra and ventral tegmental area (SN-VTA) mask on the neuromelanin-sensitive MRI (NM-MRI) template [a]

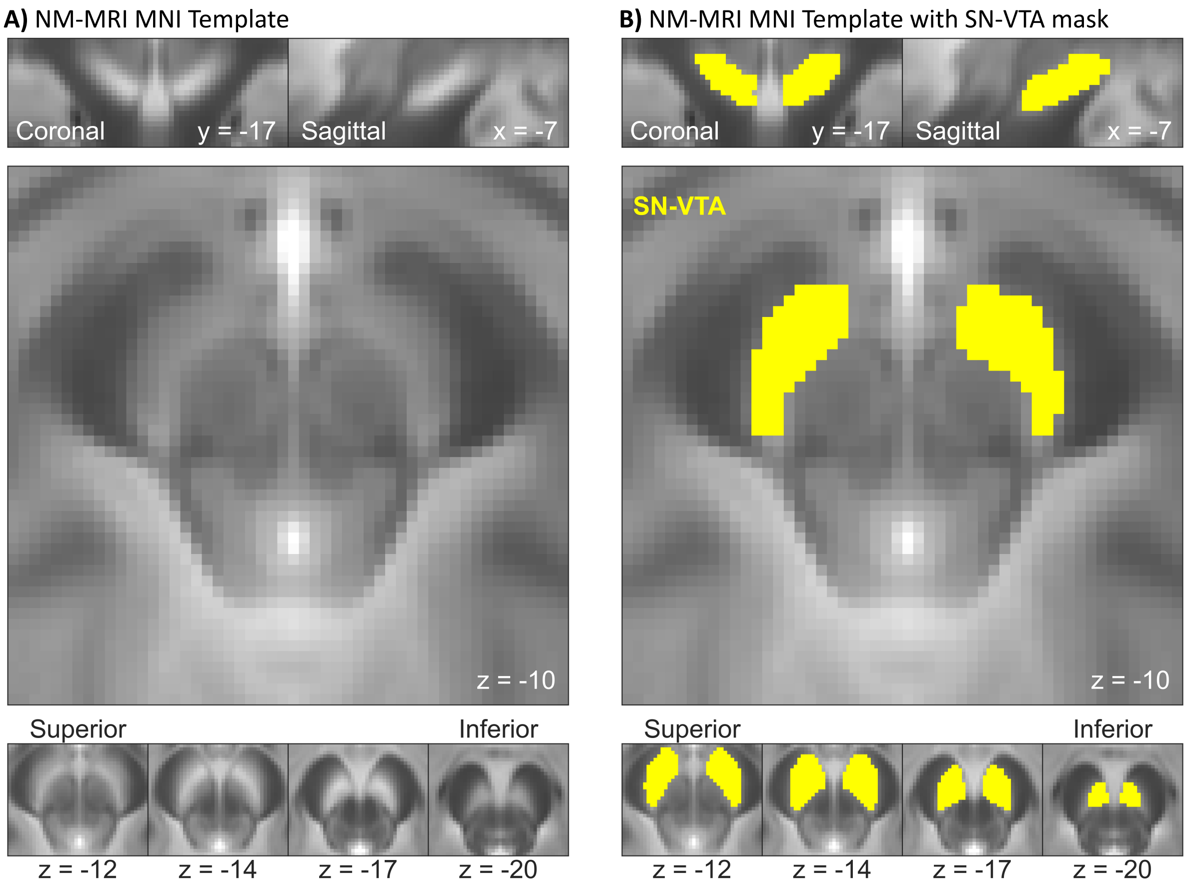

[a] Panel a displays the study NM-MRI template generated by averaging NM-MRI images normalized to the Montreal Neurological Imaging (MNI) space. Panel b shows the SN-VTA mask (yellow) on this template.

Table S1. Results from the robust linear model built to predict substantia nigra and ventral tegmental area (SN-VTA) magnetic susceptibility (ppb) with case-control status, potential clinical confounders, SN-VTA mean diffusivity (10^5 mm^2/s), and NM-CNR (controls N=38; schizophrenia N=61) [a]

Variable	Coefficient	Standard Error	t-score	p-value	[95% CI]	
(Intercept)	90.44	61.74	1.46	0.14	-30.57	211.45
Schizophrenia Group Status	-14.28	4.23	-3.38	<0.001	-22.56	-5.99
Current Smoker	0.69	5.01	0.14	0.891	-9.13	10.50
Past Smoker	-5.14	5.81	-0.88	0.377	-16.52	6.25
THC-positive UDS	-2.85	4.90	-0.58	0.561	-12.46	6.76
Male Sex	-0.16	4.61	-0.04	0.972	-9.20	8.88
Age	1.08	0.32	3.36	<0.001	0.45	1.72
SN-VTA Mean Diffusivity	0.57	0.67	0.85	0.394	-0.74	1.89
SN-VTA NM-CNR	-224.52	94.25	-2.38	0.017	-409.24	-39.81

[a] ppb=parts per billion; CI=confidence interval; THC=Delta-9-tetrahydrocannabinol; UDS=Urine drug screen; NM-CNR=Neuromelanin-sensitive MRI contrast-to-noise ratio.

Figure S3. Correlation between substantia nigra and ventral tegmental area (SN-VTA) magnetic susceptibility and age across all participants [a]

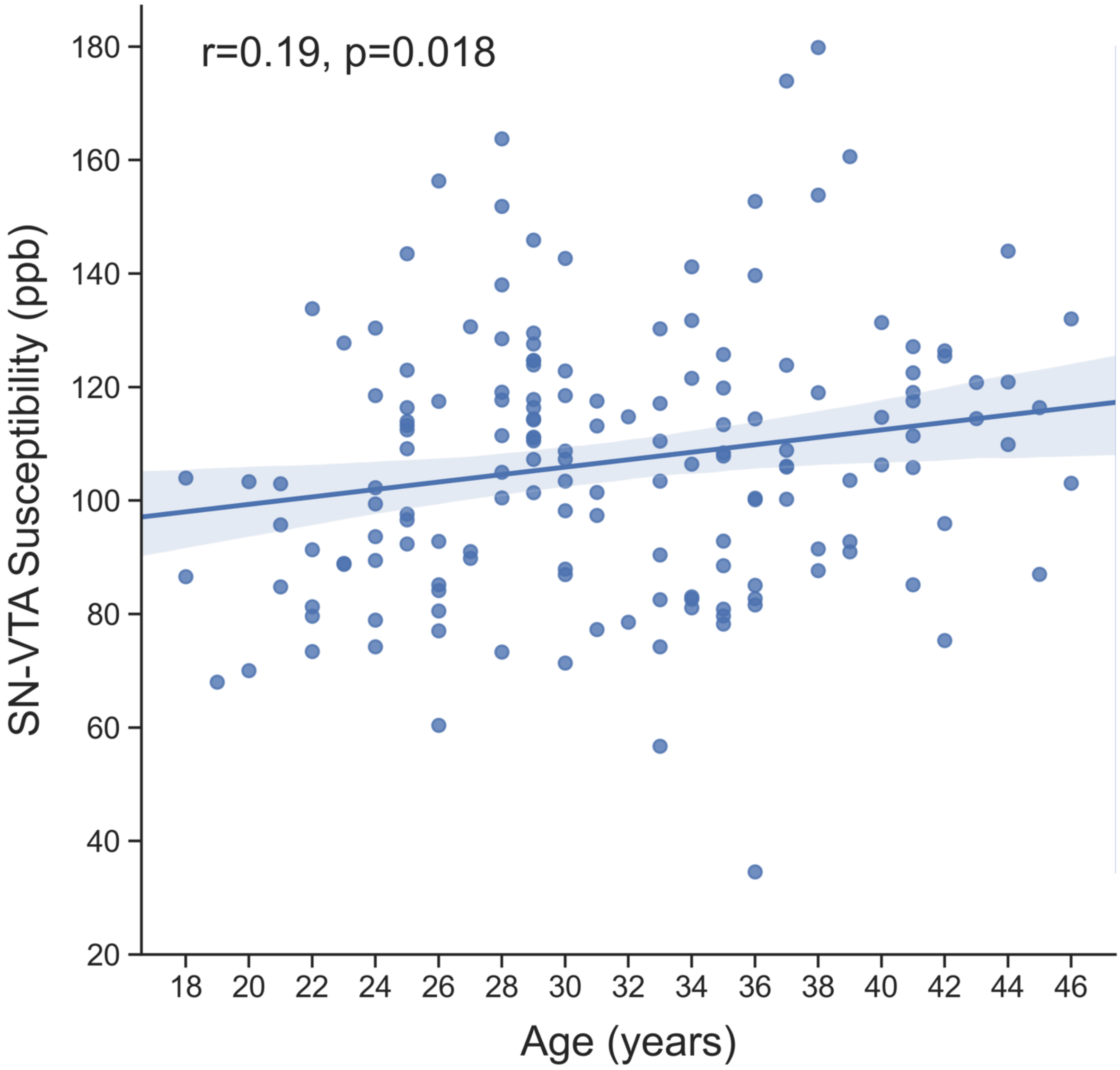

[a] ppb=parts per billion.

Figure S4. Relationship between substantia nigra and ventral tegmental area (SN-VTA) magnetic susceptibility and NM-CNR in patients with schizophrenia and controls [a]

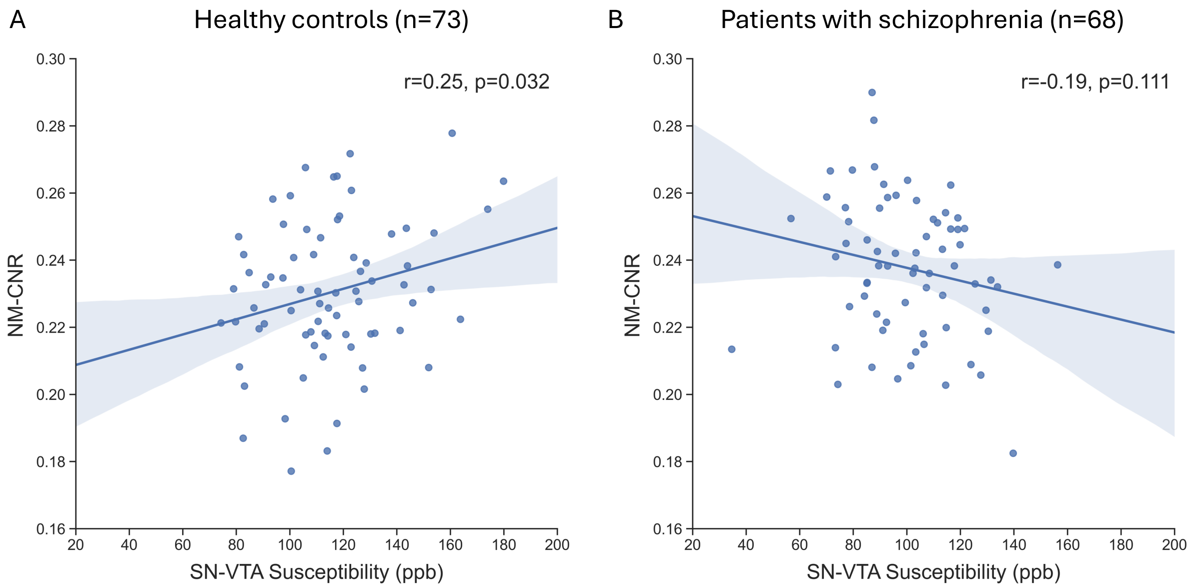

[a] Panel a displays the association between SN-VTA magnetic susceptibility, calculated by quantitative susceptibility mapping, and SN-VTA neuromelanin-sensitive MRI contrast-to-noise ratio (NM-CNR) in healthy controls. In panel b, the results from the same analysis is shown in patients with schizophrenia. There was a significant difference between these correlation coefficients (z=2.6, p=0.009). ppb=parts per billion.

Figure S5. Substantia nigra and ventral tegmental area (SN-VTA) quantitative susceptibility mapping (QSM) analysis comparing unmedicated with medicated patients [a]

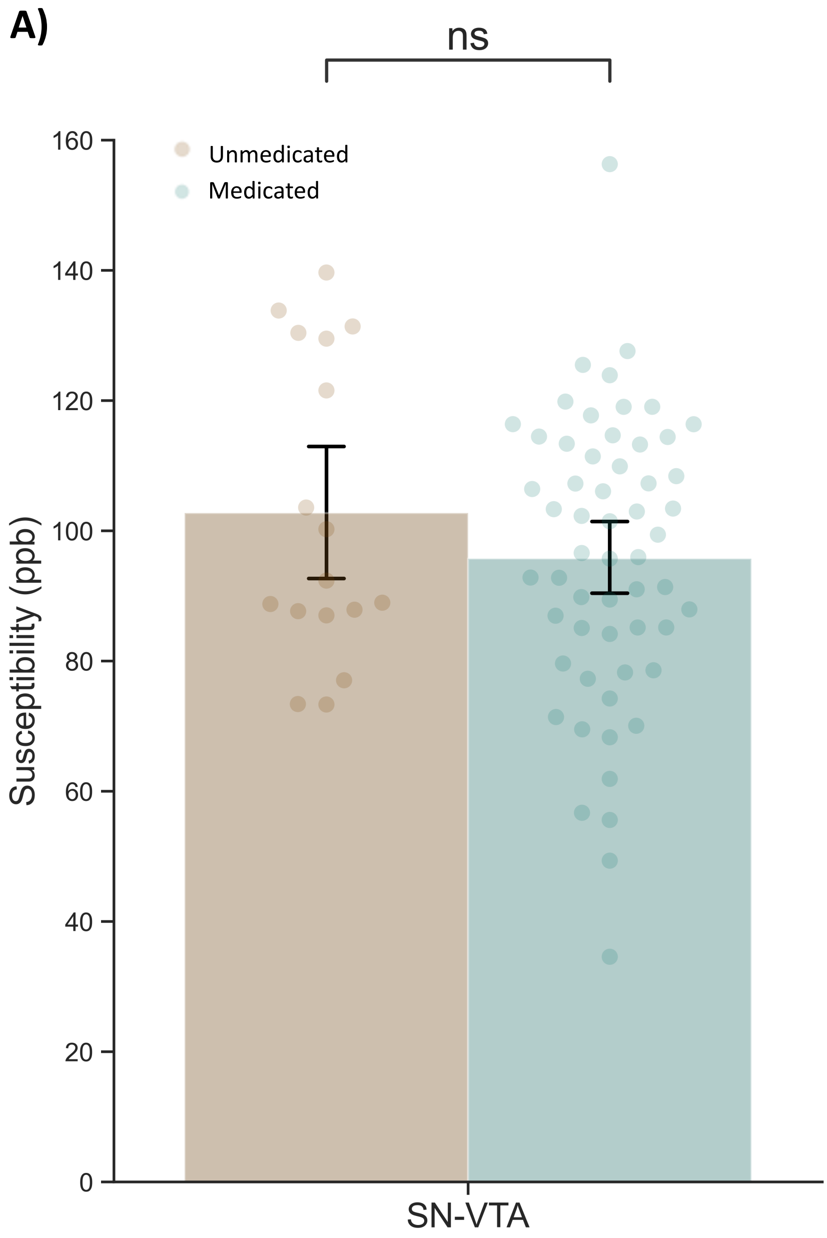

[a] Bar chart showing mean SN-VTA susceptibility, calculated by QSM, for the unmedicated patients (brown) unmedicated and those taking an antipsychotic (medicated; green) (95% confidence intervals) with values also plotted for each participant. Susceptibility was not significantly different between the groups (t=-1.13, p=0.262). ns=non-significant (p>0.05). ppb=parts per billion.

Figure S6. Correlating substantia nigra and ventral tegmental area (SN-VTA) magnetic susceptibility with clinical variables [a]

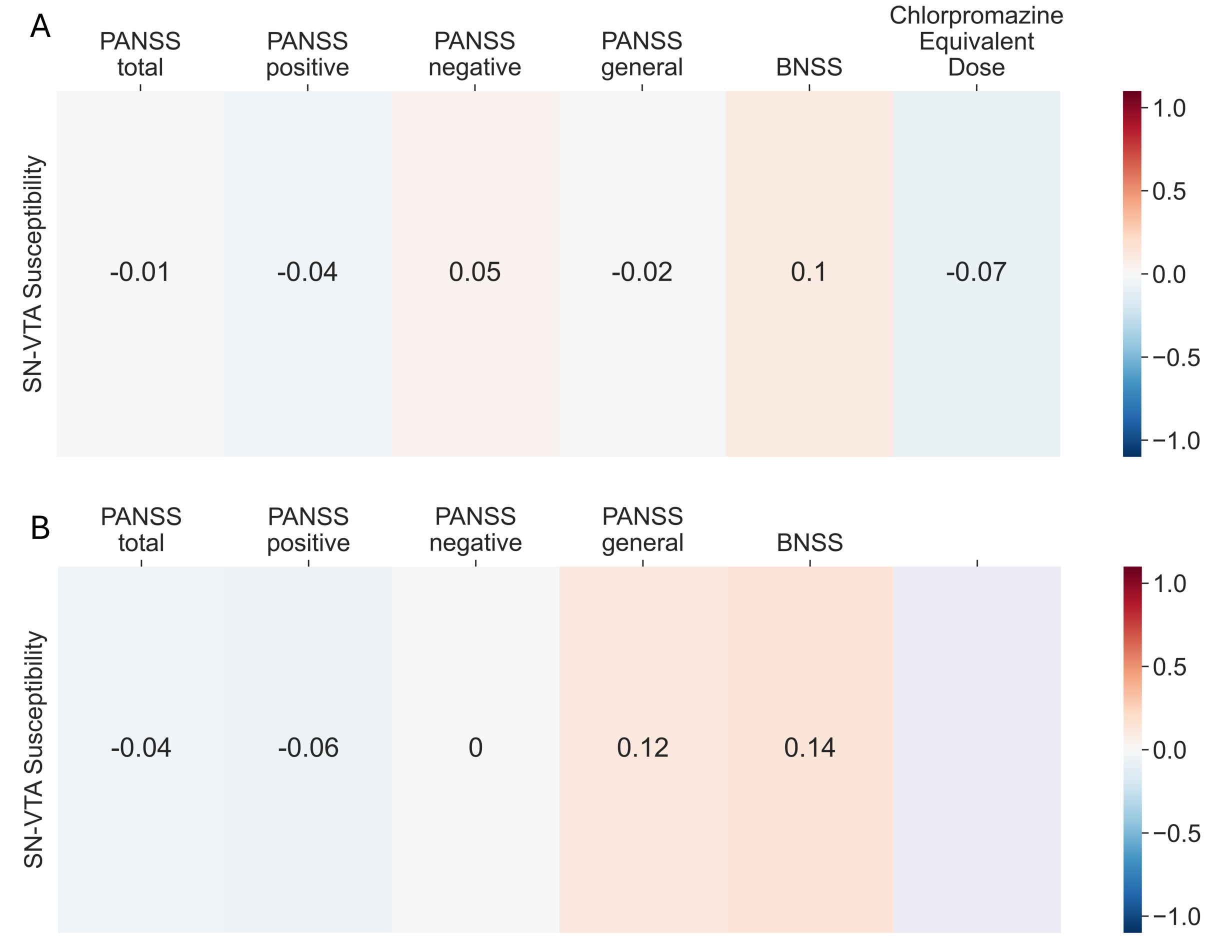

[a] Panel a is a heatmap displaying the Pearson's r correlation between magnetic susceptibility derived from quantitative susceptibility mapping (QSM) against clinical variables in the full schizophrenia group. No correlation was statistically significant (p>0.05). Panel b is an additional heatmap showing only the correlations for the antipsychotic-free group. No correlation was statistically significant (p>0.05). PANSS=Positive and Negative Syndrome Scale; BNSS=Brief Negative Symptoms Scale; SN-VTA=substantia nigra and ventral tegmental area.

Figure S7. Voxelwise substantia nigra and ventral tegmental area (SN-VTA) quantitative susceptibility mapping (QSM) case-control analysis with the spatial extent method [a]

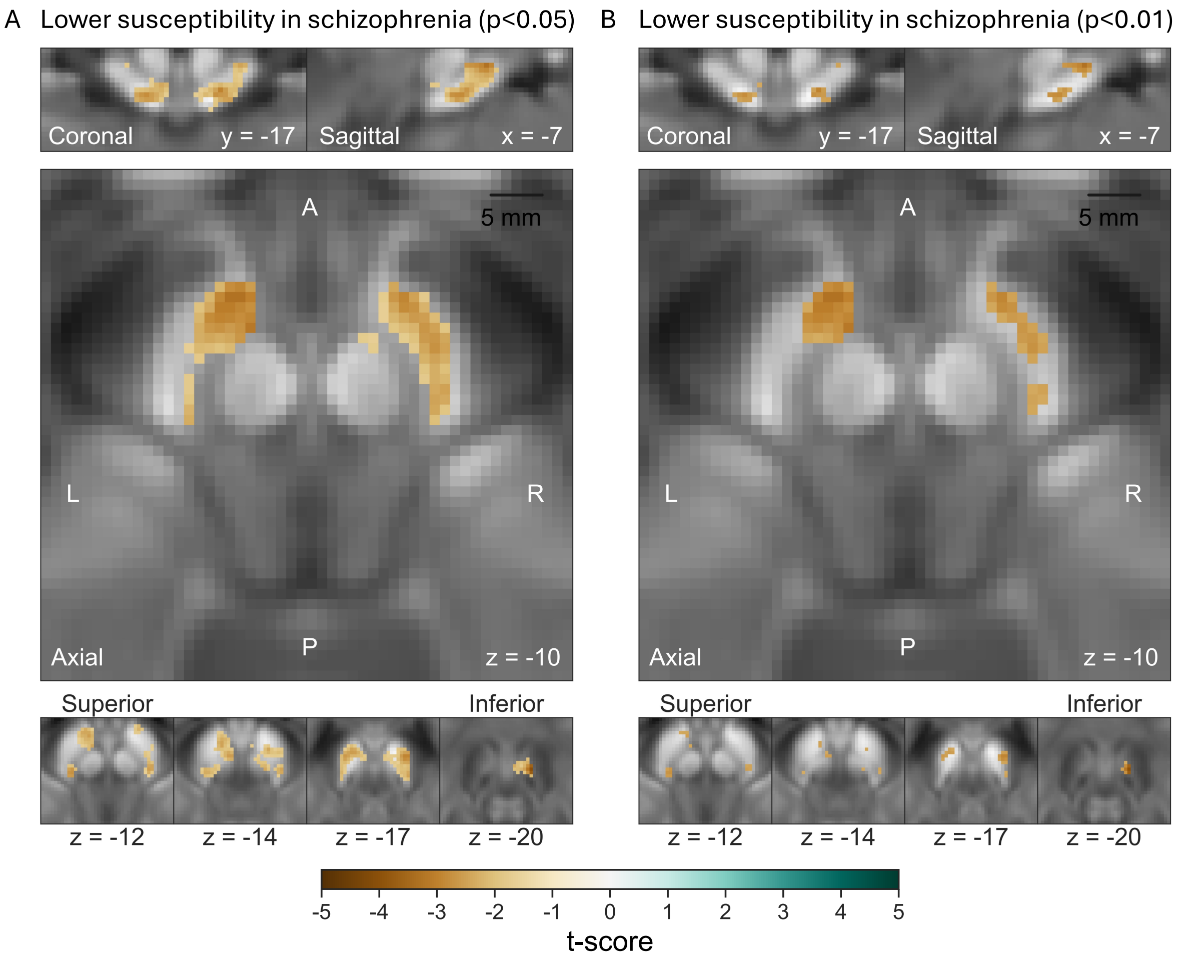

[a] Panel a displays the 794 out of the 1790 SN-VTA voxels where schizophrenia was associated with significantly lower susceptibility at a one-sided threshold of p<0.05. The spatial extent of this effect was significant when compared to a null distribution from 10,000 random permutations of the case-control status (p<0.001). The colorbar refers to the t-score, where brown indicates lower susceptibility in schizophrenia relative to controls and green lower susceptibility in controls. Schizophrenia was associated with greater susceptibility in 2 voxels at a one-sided threshold p<0.05 but this was not significant on permutation testing (p=1). Panel b shows the 217 voxels with lower susceptibility in

schizophrenia at a one-sided threshold $p<0.01$. These were significant on permutation testing ($p<0.001$). Schizophrenia was not associated with higher susceptibility in any voxels at this threshold.

Figure S8. Relationship between substantia nigra and ventral tegmental area (SN-VTA) voxel magnetic susceptibility and Positive and Negative Syndrome Scale (PANSS) positive score [a]

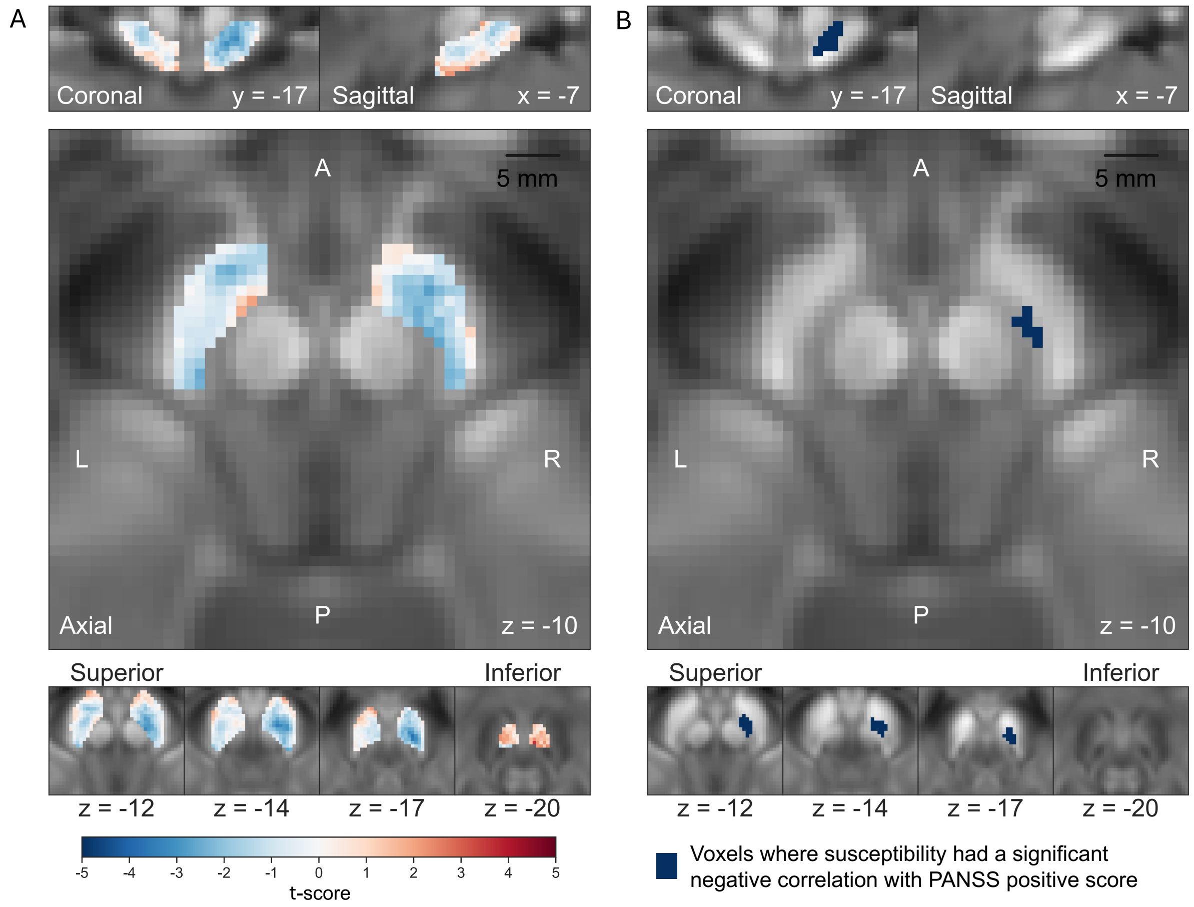

[a] Panel a shows the SN-VTA t-score map. The colorbar refers to the t-score, where red indicates a direct relationship between magnetic susceptibility and PANSS positive score and blue indicates an indirect association between these variables. The peak t-score was in the right medial SN-VTA (x=7, y=-18, z=−15; t=-3.85). Panel b displays a single cluster where susceptibility had a significant negative correlation with PANSS positive score (threshold-free cluster enhancement, Benjamini-Hochberg corrected p<0.05). No clusters were identified where susceptibility positively correlated with PANSS positive score.

Figure S9. Correlation between dopamine synthesis capacity (K_i^{cer}) and susceptibility across regions of interest [a]

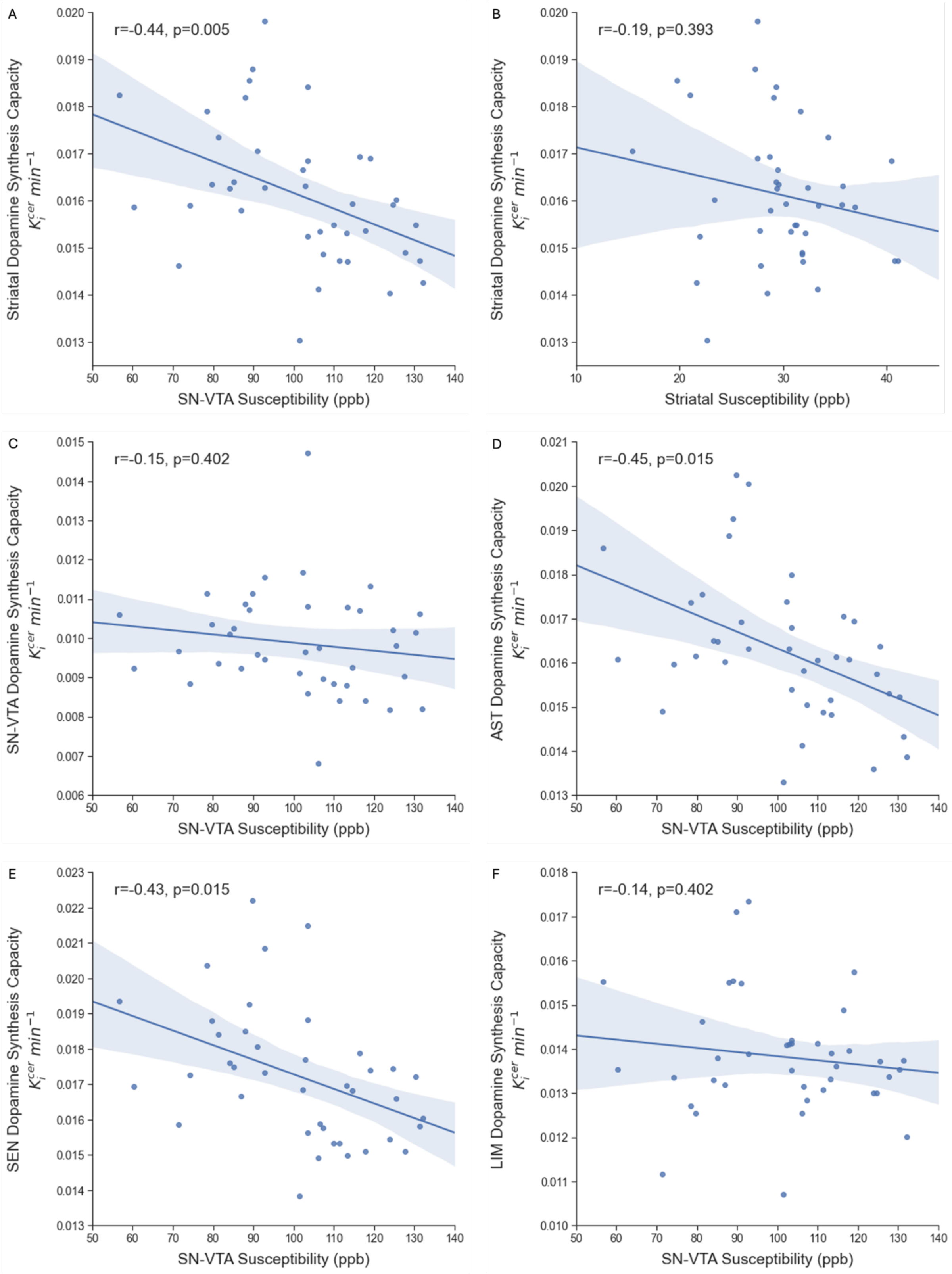

[a] Panel A shows substantia nigra and ventral tegmental area (SN-VTA) susceptibility with striatal K_i^{cer}, panel B striatal susceptibility with striatal K_i^{cer}, and panel C SN-VTA susceptibility with SN-VTA K_i^{cer}. SN-VTA susceptibility was correlated with associative (AST) striatal K_i^{cer} in panel D, sensorimotor (SEN) striatal K_i^{cer} in panel E, and limbic (LIM) striatal K_i^{cer} in panel F. K_i^{cer}=influx constant for [18F]-DOPA uptake in the region of interest relative to the control region (cerebellum); ppb=parts per billion.

Table S2. Results from the robust linear model built to predict striatal dopamine synthesis capacity (10^3 K_i^{cer} min^{-1}) with substantia nigra and ventral tegmental area (SN-VTA) magnetic susceptibility (ppb), potential clinical confounders, SN-VTA mean diffusivity (10^5 mm^2/s) and NM-CNR (schizophrenia N=33) [a]

Variable	Coefficient	Standard Error	t-score	p-value	[95% CI]	
(Intercept)	21.710	4.978	4.36	<0.001	11.954	31.467
SN-VTA Susceptibility	-0.021	0.007	-2.88	0.004	-0.035	-0.007
SN-VTA NM-CNR	16.053	7.064	2.27	0.023	2.207	29.899
SN-VTA Mean Diffusivity	-0.051	0.052	-0.98	0.328	-0.153	0.051
Current Smoker	-0.909	0.324	-2.81	0.005	-1.544	-0.274
Past Smoker	-0.447	0.405	-1.10	0.27	-1.241	0.347
THC-positive UDS	0.872	0.328	2.66	0.008	0.230	1.514
Male Sex	-1.422	0.300	-4.74	<0.001	-2.010	-0.834
Age	-0.070	0.022	-3.26	<0.001	-0.113	-0.028

[a] ppb=parts per billion; SN-VTA=substantia nigra and ventral tegmental area; NM-CNR=neuromelanin-sensitive MRI contrast-to-noise ratio; CI=confidence interval; THC=Delta-9-tetrahydrocannabinol; UDS=urine drug screen.

Supplementary Methods

Common study exclusion criteria

Scans were only completed for female participants if they provided a negative urine pregnancy test. Breastfeeding volunteers were excluded. Common exclusion criteria for both groups included a history of oncological, neurological, endocrine, or other significant medical conditions (minor conditions, such as well-controlled asthma, were permitted if judged unlikely to affect measures) and, for participants undergoing a positron emission tomography (PET) scan, if previous radiation exposure would mean the participant was exposed to over 10 mSv of radiation in the last 12 months by taking part. Participants were excluded if a urinary drug screen (UDS) was positive for any of the following: cocaine, opiates, methadone, amphetamine, methamphetamine, barbiturates, 3,4-methylenedioxy methamphetamine (MDMA), benzodiazepine, or phencyclidine.

Smoking status

Smoking status was assessed using an interview-based questionnaire, following previously established criteria (1). If participants had never smoked at least 5 cigarettes per week for a duration of 3 months or longer they were defined as having never smoked. If they were currently smoking at this level, they were characterized as being a current smoker. Former

smokers were individuals who had previously met this criterion but were no longer smoking regularly.

Neuromelanin-sensitive MRI (NM-MRI) Data Processing

Our NM-MRI analysis pipeline and midbrain atlas, containing the SN-VTA and crus cerebri (CC) masks, are publicly available at: https://github.com/lukevano/KCL_Neuromelanin-MRI. An NM-MRI template was generated by linearly co-registering each subject's NM-MRI image to their T1-weighted image using Advanced Normalization Tools (ANTs) 2.4.0 (2), applying the transformation-matrix and warp-field to normalize these to the Montreal Neurological Imaging (MNI) space, before averaging these images to form the template. With ITK-SNAP (3) we manually outlined the SN-VTA and crus cerebri (CC) on this template, using the images provided by Cassidy et al. as a guide (4).

For each participant, an NM-MRI contrast-to-noise ratio (NM-CNR) map was generated by applying the following equation to each voxel in the neuromelanin-sensitive MRI image:

(voxel intensity - mode crus cerebri voxel intensity) / mode crus cerebri voxel intensity

We used the gaussian_kde function in the Python package SciPy v1.12.0 (5) to calculate the mode crus cerebri intensity for the equation above. Each NM-CNR image was normalized to the MNI space, using the steps outlined above.

To remove voxels that were less reliable at capturing the SN-VTA we eroded mask edge voxels prone to extreme values (having an NM-CNR beyond the 1st or 99th percentile for a given participant across more than three participants) or consistently low signal (NM-CNR under 0.05 in over 90% of participants).

QSM Data Processing

We ran a post-processing pipeline to generate QSM images used by previous studies (6, 7). A brain mask was generated from the first echo time magnitude image of the 3D gradient recall echo (GRE) using the FMRIB Software Library (FSL) Brain Extraction Tool (BET) (8). Using the Fit_ppm_complex.m function of the MEDI toolbox, the frequency shift for each voxel was calculated from the phase images of all echo times (9). We then calculated the local frequency shift from the frequency shifts of all voxels within the generated brain mask, eroded to approximately 95% in size, using the projection onto dipole fields method (10). The iterative Tikhonov dipole inversion method (11) was then employed to calculate the final quantitative susceptibility maps from the local frequency shifts.

Given that GRE is prone to distortion at air/bone boundaries, non-linear realignment of the GRE magnitude brain from the first echo to the T1-weighted brain was carried out with a low 15mm resolution spline grid using the NiftyReg toolbox to only correct distortions caused by the large B0 inhomogeneities at these boundaries (12). The subject T1-weighted images were spatially normalized to MNI space with non-linear co-registration using ANTs.

The QSM images were normalized to Montreal Neurological Imaging (MNI) space by applying the relevant affine transformation-matrices and warp-fields generated from this prior step and the coregistration of the T1-weighted brain to the MNI space. These normalized QSM images were spatially smoothed with a 1-mm full-width-at-half maximum Gaussian kernel prior to voxelwise analysis.

Investigators (LJV and JS) used consensus recommendations to quality control the normalized and original QSM images (13) while blinded to participant identification. This quality control involved excluding scans with streaking or shadowing artifacts (13) and failed brain masking on visual inspection.

DTI processing

We corrected the effects of eddy currents, distortion, and head movement on the DTI data using FSL's FMRIB Software Laboratory diffusion toolbox (14, 15). Following brain extraction a diffusion tensor model was fitted using dtifit (14). We carried non-linearly co-registered each subject's fractional anisotropy (FA) image to the standard FMRIB58_FA_1mm template using FSL's tract-based spatial statistics (TBSS) toolbox (16). As our QSM voxelwise analyses were completed in the MNI152NLin2009cSym space we also non-linearly co-registered the MNI152NLin2009cSym brain to the MNI152 sixth-gen T1-w template. If SN-VTA voxels of significant case-control difference were identified, we transformed these into each subject's

DTI space for mean diffusivity analysis using the relevant affine transformation-matrices and warp-fields.

PET Data Processing

Given that food and smoking (17) can affect dopamine synthesis capacity, participants fasted for 6 hours and avoided smoking for 4 hours before the scan. An MR localizer was used to center the field of view on the thalamus. A total of 95 minutes of emission data were acquired. Data were rebinned into 26 time frames comprising 30 seconds of background frame, four 60-second frames, three 120-second frames, three 180-second frames, and fifteen 300-second frames. We used a 3D re-projection algorithm to reconstruct the data (18).

We used the Patlak-Gjedde method for a standard graphical approach (18) using in-house script incorporating Matlab packages (Piwave (19), FDOPA package), and Statistical Parametric Mapping (SPM) 12 (20). As previously described, head movement was corrected by realigning each frame to the frame at 15 minutes (21). We generated individual mean motion-corrected dynamic images by summing the realigned frames. We linearly co-registered each participant's PET summed image to their T1-weighted image using ANTs. The same striatal subdivision atlas (22), which also contains a cerebellar mask, and SN-VTA mask used for the QSM processing steps were moved from the MNI space to each

participant’s PET space by applying the inverse of the relevant warp-fields and transformation-matrices using ANTs.

PET data underwent quality control for non-physiological parameter estimates (negative or out of physiological range), uncorrected anatomical miss-segmentation, and excessed of motion correction, by previously outlined steps (21).

<u>Identifying voxels of significant case-control susceptibility difference using the spatial extent method</u>

We applied the spatial extent method (23) to identify voxels showing significant case-control differences in susceptibility, consistent with prior NM-MRI studies investigating the SN-VTA (4, 24). This approach utilized the t-score map generated from the robust linear regression, where voxel susceptibility was predicted from case-control status, controlling for clinical confounders. Voxels with lower susceptibility in schizophrenia were identified using a one-sided threshold of $p<0.05$. To detect voxels with higher susceptibility, we reversed the operation.

We tested hypotheses by summing the number of voxels where $p<0.05$ (spatial extent of the effect) and comparing this to a null distribution from 10,000 random permutations of the case-control status (4, 24). The same procedure was also applied for voxels identified at

a one-sided p<0.01. These significant voxels were illustrated on the QSM template in MNI space in the supplementary.

References

1. Elbejjani M, Auer R, Jacobs DR, et al.: Cigarette smoking and gray matter brain volumes in middle age adults: the CARDIA Brain MRI sub-study. Transl Psychiatry 2019; 9:78
2. Avants B, Tustison NJ, Song G: Advanced Normalization Tools: V1.0 [Internet]. The Insight Journal 2009; [cited 2023 Oct 3] Available from: https://www.insight-journal.org/browse/publication/681
3. Yushkevich PA, Piven J, Hazlett HC, et al.: User-guided 3D active contour segmentation of anatomical structures: significantly improved efficiency and reliability. Neuroimage 2006; 31:1116–1128
4. Cassidy CM, Zucca FA, Girgis RR, et al.: Neuromelanin-sensitive MRI as a noninvasive proxy measure of dopamine function in the human brain. Proc Natl Acad Sci U S A 2019; 116:5108–5117
5. Virtanen P, Gommers R, Oliphant TE, et al.: SciPy 1.0: fundamental algorithms for scientific computing in Python. Nat Methods 2020; 17:261–272
6. Lorio S, Sedlacik J, So P-W, et al.: Quantitative MRI susceptibility mapping reveals cortical signatures of changes in iron, calcium and zinc in malformations of cortical development in children with drug-resistant epilepsy. Neuroimage 2021; 238:118102
7. Tortora D, Severino M, Sedlacik J, et al.: Quantitative susceptibility map analysis in preterm neonates with germinal matrix-intraventricular hemorrhage. J Magn Reson Imaging 2018; 48:1199–1207
8. Smith SM: Fast robust automated brain extraction. Hum Brain Mapp 2002; 17:143–155
9. Liu T, Wisnieff C, Lou M, et al.: Nonlinear formulation of the magnetic field to source relationship for robust quantitative susceptibility mapping. Magnetic Resonance in Medicine 2013; 69:467–476
10. Liu T, Khalidov I, de Rochefort L, et al.: A novel background field removal method for MRI using projection onto dipole fields (PDF). NMR Biomed 2011; 24:1129–1136

11. Karsa A, Punwani S, Shmueli K: An optimized and highly repeatable MRI acquisition and processing pipeline for quantitative susceptibility mapping in the head-and-neck region. Magnetic Resonance in Medicine 2020; 84:3206–3222

12. Modat M, Cash DM, Daga P, et al.: Global image registration using a symmetric block-matching approach. J Med Imaging (Bellingham) 2014; 1:024003

13. QSM Consensus Organization Committee, Bilgic B, Costagli M, et al.: Recommended implementation of quantitative susceptibility mapping for clinical research in the brain: A consensus of the ISMRM electro-magnetic tissue properties study group. Magn Reson Med 2024; 91:1834–1862

14. Jenkinson M, Beckmann CF, Behrens TEJ, et al.: FSL. NeuroImage 2012; 62:782–790

15. Andersson JLR, Sotiropoulos SN: An integrated approach to correction for off-resonance effects and subject movement in diffusion MR imaging. Neuroimage 2016; 125:1063–1078

16. Smith SM, Jenkinson M, Johansen-Berg H, et al.: Tract-based spatial statistics: Voxelwise analysis of multi-subject diffusion data. NeuroImage 2006; 31:1487–1505

17. Bloomfield MA, Pepper F, Egerton A, et al.: Dopamine Function in Cigarette Smokers: An [18F]-DOPA PET Study. Neuropsychopharmacology 2014; 39:2397–2404

18. Egerton A, Demjaha A, McGuire P, et al.: The test-retest reliability of 18F-DOPA PET in assessing striatal and extrastriatal presynaptic dopaminergic function. Neuroimage 2010; 50:524–531

19. Turkheimer FE, Brett M, Visvikis D, et al.: Multiresolution analysis of emission tomography images in the wavelet domain. J Cereb Blood Flow Metab 1999; 19:1189–1208

20. Ashburner J: SPM: a history. Neuroimage 2012; 62:791–800

21. Nordio G, Easmin R, Giacomel A, et al.: An automatic analysis framework for FDOPA PET neuroimaging. J Cereb Blood Flow Metab 2023; 43:1285–1300

22. Martinez D, Slifstein M, Broft A, et al.: Imaging human mesolimbic dopamine transmission with positron emission tomography. Part II: amphetamine-induced dopamine release in the functional subdivisions of the striatum. J Cereb Blood Flow Metab 2003; 23:285–300

23. Friston KJ, Worsley KJ, Frackowiak RS, et al.: Assessing the significance of focal activations using their spatial extent. Hum Brain Mapp 1994; 1:210–220

24. Vano LJ, McCutcheon RA, Rutigliano G, et al.: Mesostriatal Dopaminergic Circuit Dysfunction in Schizophrenia: A Multimodal Neuromelanin-Sensitive Magnetic Resonance Imaging and [18F]-DOPA Positron Emission Tomography Study. Biological Psychiatry 2024; 96:674–683

1
